# Assessment of patient perspectives in the CONVINCE trial in end-stage kidney disease - a domain-guided approach

**DOI:** 10.3389/fneph.2026.1767603

**Published:** 2026-08-26

**Authors:** Kathrin I. Fischer, Krister Cromm, Peter J. Blankestijn, Bernard Canaud, Jörgen Hegbrant, Claudia Barth, Mark Woodward, Giovanni F. M. Strippoli, Andrew Davenport, Michiel L. Bots, Matthias Rose

**Affiliations:** 1 Charité – Universitätsmedizin Berlin, Corporate Member of Freie Universität Berlin and Humboldt Universität zu Berlin, Medizinische Klinik mit Schwerpunkt für Psychosomatik, Center for Patient-Centered Outcomes Research, Berlin, Germany; 2 Global Medical Office, Fresenius Medical Care Deutschland GmbH, Bad Homburg, Germany; 3 Department of Nephrology and Hypertension, University Medical Center Utrecht, Utrecht, Netherlands; 4 School of Medicine, Montpellier University, Montpellier, France; 5 Division of Nephrology, Department of Clinical Sciences, Lund University, Lund, Sweden; 6 B. Braun Avitum AG, Medical Scientific Affairs, Melsungen, Germany; 7 The George Institute for Global Health, Imperial Collage London, London, United Kingdom; 8 The George Institute for Global Health, University of New South Wales, Sydney, NSW, Australia; 9 Department of Precision and Regenerative Medicine and Ionian Area (DIMEPRE-J), University of Bari, Bari, Italy; 10 University of Sydney, School of Public Health, Sydney, Australia; 11 University College London (UCL) Department of Renal Medicine, Royal Free Hospital, Division of Medicine, University College London, London, United Kingdom; 12 Julius Center for Health Sciences and Primary Care, University Medical Center Utrecht, Utrecht University, Utrecht, Netherlands

**Keywords:** end-stage kidney disease, maintenance dialysis therapy, patient perspective, patient-reported outcome measures, qualitative research, quality of life

## Abstract

**Background:**

Patient-reported outcome (PRO) measures are increasingly used in clinical trials and clinical care to assess symptoms, functioning, and health-related quality of life (HRQoL). In dialysis research, instrument-driven approaches that rely on legacy measures may not fully capture outcomes that matter most to patients or treatment-related experiences such as dialysis-specific fatigue and recovery. Therefore, a patient-informed, domain-guided PRO toolbox for adults with end-stage kidney disease (ESKD) receiving maintenance dialysis was developed and applied in the CONVINCE trial.

**Methods:**

A multi-method, triangulation approach was used, comprising: 1) a targeted literature review of PROMs and symptom concepts used in maintenance dialysis research, 2) review of international core outcome initiatives relevant to kidney disease and haemodialysis, 3) semi-structured interviews and focus groups with patients (*n* = 13) and health care professionals (*n* = 4). Concepts identified across sources were compiled, compared, and prioritized based on recurrence across evidence sources, salience to patients, anticipated sensitivity to dialysis modality, and feasibility of repeated assessment within the CONVINCE trial.

**Results:**

The literature review identified 97 PROMs. Domains and symptom content were extracted from these instruments and mapped against concepts covered by international core outcome initiatives and qualitative data. Triangulation resulted in an ESKD PRO toolbox comprising nine prioritized health domains: general health/health perception, fatigue, physical function, social roles, depression, anxiety, pain, sleep, and cognitive function. Most of these domains could be assessed within the PROMIS^®^ health domain framework. To complement these generic domain measures, a dialysis-specific symptom list and additional items to assess dialysis-related fatigue, treatment-related recovery time were included.

**Conclusion:**

The ESKD PRO toolbox provides a patient-informed, domain-guided framework for repeated longitudinal assessment of outcomes relevant to patients receiving maintenance dialysis. Its application in CONVINCE illustrates how PROMIS^®^ -based domain assessment can be combined with dialysis-specific symptom and fatigue measures to capture treatment-related patient experiences.

## 1 Introduction

Patients with end-stage kidney disease (ESKD) undergoing kidney replacement therapy have high mortality rates and an increased burden of disease (1). The prevalence and incidence of ESKD is rising along with its costs, leading to a serious public health issue (2, 3). As kidney transplantation is only available for selected patients, maintenance dialysis therapy is inevitable for the majority of ESKD patients (4). Peritoneal dialysis, haemodialysis (HD) and hemodiafiltration (HDF) are established therapies, with most patients, world-wide, currently treated with HD (4).

Health-related quality of life (HRQL) of ESKD patients is negatively affected by symptoms and limitations in functional status (5–9). Moreover, HRQL is a valuable outcome indicator, especially since poor HRQL is associated with increased mortality (10). Besides the impact of ESKD on HRQL, there is the additional impact of regular dialysis therapy on HRQL. Patients report a negative effect on emotional wellbeing based on the disease and the additional dependency on dialysis and being tied to a HD machine with a fixed treatment schedule due to logistical constraints (11). Some patients report impaired cognitive and physical functioning especially on the days of the HD treatment (11). Post-dialysis fatigue is frequently reported by patients, which has a detrimental effect on their HRQL, including their physical activity levels as indicated by a decrease in the number of daily steps (12). Thus, HRQL of patients is an important outcome measure for both patients and health providers, in addition to morbidity and mortality.

Using patient-reported outcome measurements (PROMs) to assess HRQL and symptoms is becoming increasingly requested to evaluate therapies, not only in clinical trials and for notified bodies including the American Food and Drug Administration (FDA) and the European Medicines Agency (EMA) to approve therapies but also for clinical practice to assess patient experience (13–15). As defined by the FDA, a patient-reported outcome (PRO) is “a measurement based on a report that comes directly from the patient about the status of a patient’s health condition without amendment or interpretation of the patient’s response by a clinician or anyone else” (14). To appropriately include patients’ perspectives in routine clinical care and in clinical research, careful consideration is needed when choosing PROs and respective PROMs. Aspects such as the conceptual model, the measurement model, validity, reliability, and responsiveness, translation, interpretability of scores and the burden for participants and investigators have to be considered when developing a patient-centred outcome strategy in clinical research and practice (16).

The CONVINCE trial was a European, multi-centre, prospective, randomized controlled trial aiming to compare high-dose HDF with high-flux HD with a primary outcome of all-cause-mortality (17). An important secondary objective of the study was to assess HRQL, and potential differences between the two dialysis modalities from the patient’s perspective. Recent consensus recommendations from the EuDial Working Group on high-flux HD and HDF further underscore the importance of evaluating patient-reported and health-related quality-of-life outcomes alongside traditional clinical endpoints in dialysis research, particularly as treatment effects may be reflected in functioning, symptoms, and broader patient experience (5). A common approach in these types of study designs is to choose a PROM which has been used in similar trials before to allow comparability of results across previous studies. The problem with this approach is that improvements of PROMs cannot be accommodated (18). In addition, important health issues for patients may be overlooked, as those may change over time with advancements in health-care potentially being inadequately addressed by conventional legacy measures often developed two or three decades earlier (19).

To address this issue, the Patient-Reported Outcome Measurement Information System (PROMIS^®^) has been launched (20). The main idea of the PROMIS^®^ initiative is to provide a health domain framework, which allows a modular assessment of key health domains (18, 20). It offers a domain-based measurement framework grounded in item response theory, with item banks and short forms that allow standardized, lower-burden assessment of physical, mental, and social health on a common metric (18). Instruments based on the PROMIS^®^ framework have been shown to provide more precise, less burdensome measures over a wider measurement range (21). A domain-guided approach is particularly relevant in kidney care because symptom burden and functional limitations may vary over time, across dialysis modalities, and in relation to treatment sessions. Recent kidney literature has highlighted the suitability of PROMIS^®^ tools for chronic kidney disease (CKD) and kidney failure because these instruments can support repeated monitoring of patient-valued symptoms and functioning while facilitating comparability across studies and settings (22, 23).

The aim of this paper is to describe the development of a specialized, patient-informed PRO toolbox for adults with ESKD receiving maintenance dialysis using a domain-guided approach. Specifically, we aimed to: (1) identify health outcomes most relevant to patients undergoing maintenance dialysis, including both broader health domains and dialysis-relevant symptoms; (2) evaluate the extent to which these prioritized domains could be assessed within the PROMIS^®^ framework; and (3) assemble a feasible PRO toolbox for repeated longitudinal use in the CONVINCE trial, supplementing PROMIS^®^ where dialysis-specific concepts required more targeted assessment.

## 2 Materials and methods

**Figure 1:**
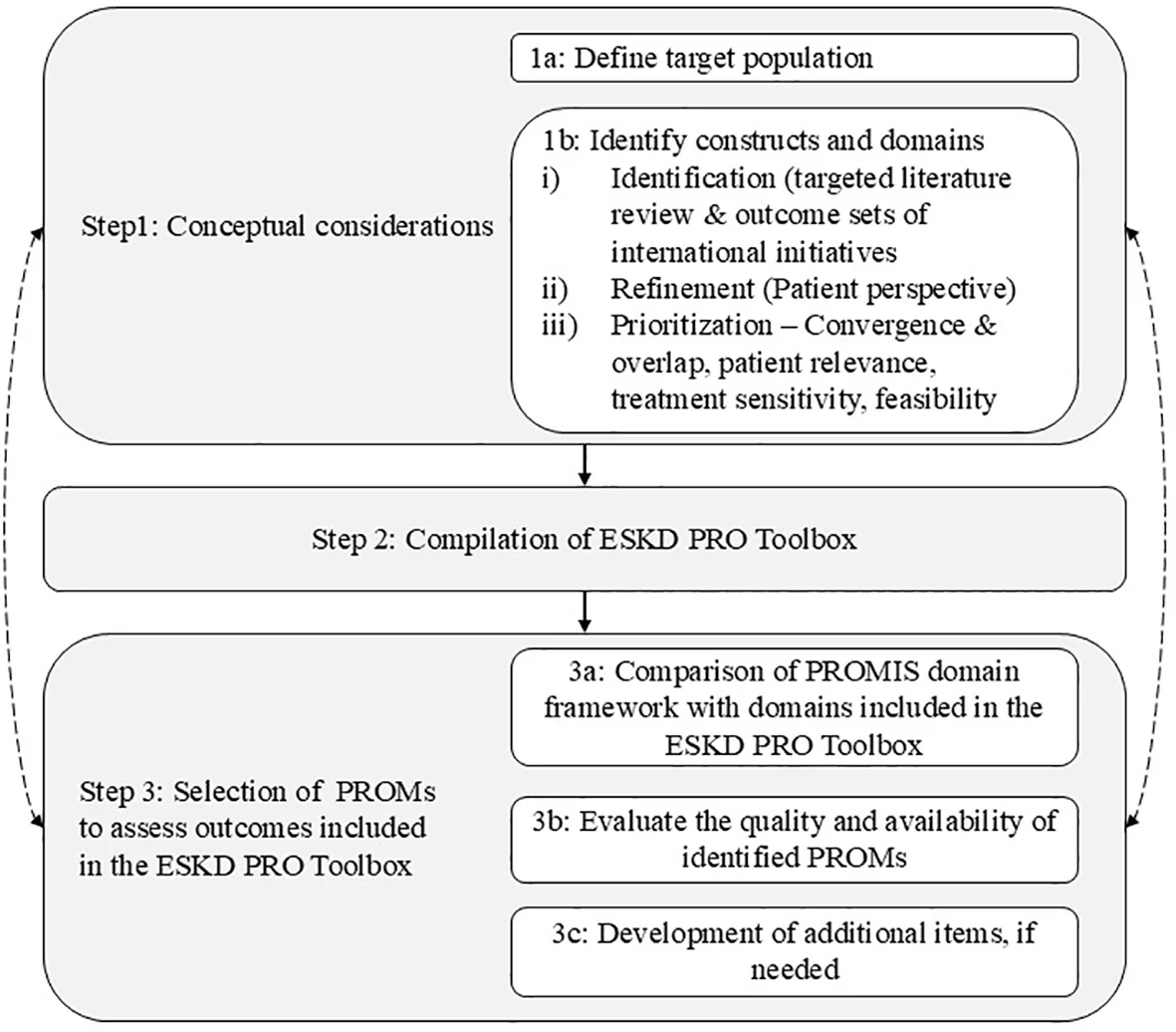
Flowchart – Iterative development steps of the ESKD PRO toolbox applied in the CONVINCE trial. PRO, Patient-reported outcome; PROMs, Patient-reported outcome measures; ESKD, end-stage kidney disease.

We identified three steps to develop our ESKD PRO toolbox. **Figure 1** summarizes the iterative development process, including (i) identification of candidate concepts from literature and outcome initiatives, (ii) refinement through patient and healthcare professional input, (iii) prioritization of concepts based on convergence, patient relevance, treatment sensitivity, and feasibility, and (iv) selection or development of instruments to assess the final set of domains and symptoms in due consideration of the PROMIS^®^ health domain framework.

### 2.1 Conceptual considerations

The conceptual framework of the ESKD PRO toolbox was determined by defining the target population and identifying health constructs and domains relevant to this population. Based on the CONVINCE trial protocol (17), we defined the target population for the ESKD PRO toolbox as adult patients (over 18 years) with ESKD undergoing maintenance dialysis therapy. The development of the ESKD PRO toolbox was informed by contemporary conceptual models of patient-reported outcomes and health-related quality of life. In particular, the PROMIS^®^ health domain framework provided the overarching domain structure because it conceptualizes health as a multidimensional construct comprising physical, mental, and social health domains. This domain-based perspective is also consistent with broader theoretical models of health outcomes, including models that differentiate symptoms, functioning, and general health perception, including the Wilson and Cleary model (24).

The subsequent identification of relevant constructs, health domains and symptoms were carried out by a multi-method approach including targeted review of the literature, review of core outcomes sets, and inclusion of patient perspectives. To facilitate conceptual clarity, we distinguish between health outcomes, constructs, health domains, and symptoms. Constructs are theoretical concepts representing broader aspects of health and wellbeing, such as health-related quality of life. Health domains are conceptually defined components of such constructs (e.g., physical function, social participation, sleep disturbance). Symptoms refer to subjective experiences of deviations from normal physical or psychological functioning, such as fatigue, pain, itching, or muscle cramps.

#### 2.1.1 Literature review

**Figure 2:**
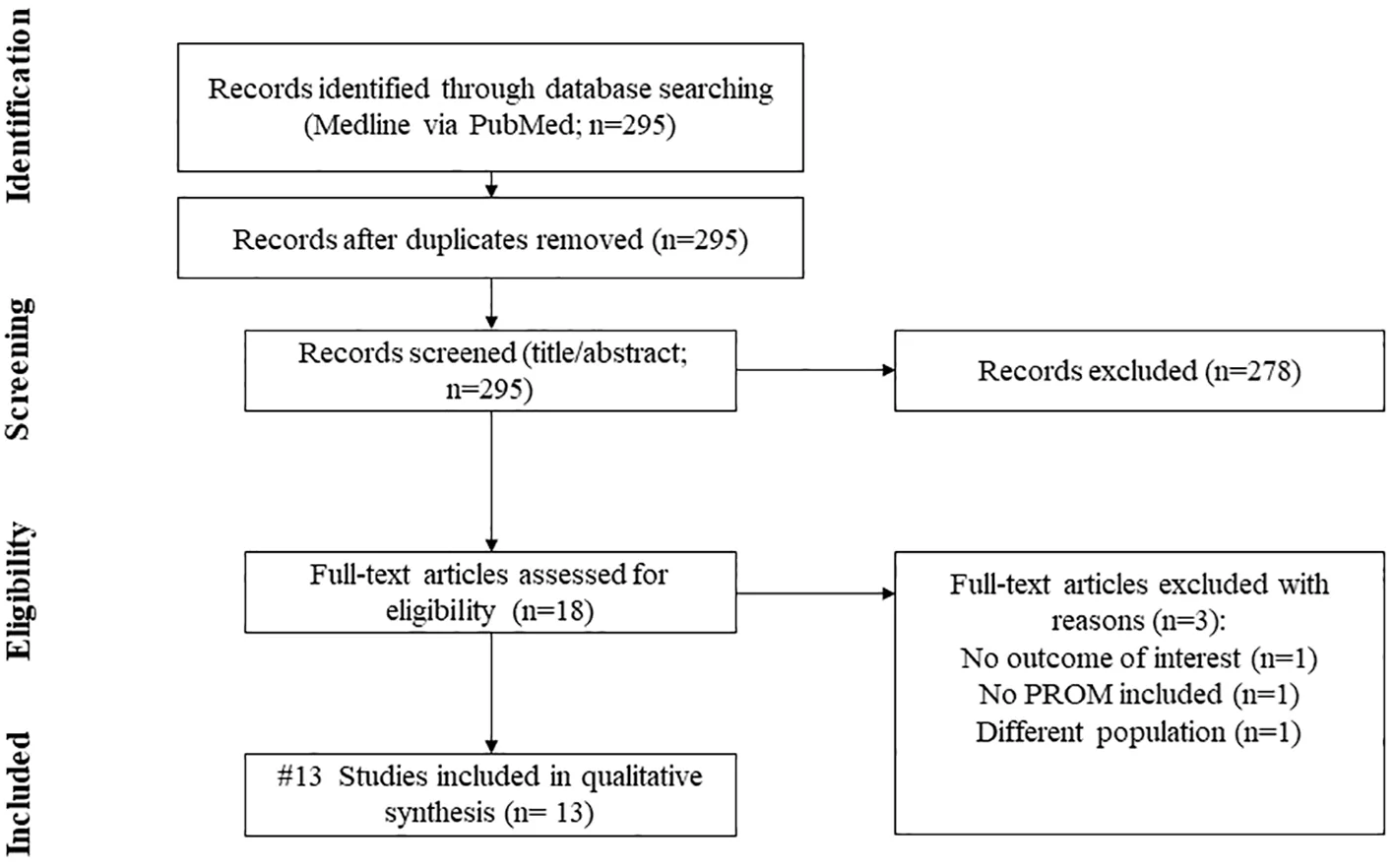
PRISMA flowchart, PROM, patient-reported outcome measure.

We reviewed the literature in terms of two different aspects. First, to identify PROMs applied in ESKD patients undergoing maintenance dialysis therapy; second, to determine symptoms related to ESKD or dialysis therapy. A targeted literature review was undertaken during trial planning to identify patient-relevant concepts and PROMs previously used among adults with ESKD receiving maintenance dialysis. Searches were conducted in Medline (via PubMed) using predefined concepts related to PROs/PROMs, ESKD, and dialysis, restricted to English-language systematic reviews published from 2000 to 2018. As shown in **Figure 2**, 295 records were identified, 18 full-text reviews were assessed, and 13 were included in the qualitative synthesis. Study selection and data extraction was perform by one reviewer, and discussed in a reconciliation meeting (further details see **Supplementary Tables 1**, **2**). For each identified PROM, domains, subscales, and symptom-specific items were reviewed. Symptoms were extracted through item-level mapping whenever symptom content was explicitly represented in questionnaire items, whereas broader health domains were mapped at the domain or subscale level.

#### 2.1.2 Outcome sets international initiatives

We used the Core Outcome Measures in Effectiveness Trials (COMET) database using the search term ‘kidney disease’ to identify international initiatives and studies focusing on development of core outcome sets in field of ESKD. Concepts derived from initiatives were compared with the literature-derived health domains and symptoms and subsequently reconciled with patient and healthcare professional interview findings. Where there was incomplete overlap, concepts supported across multiple sources and considered highly relevant to patients undergoing maintenance dialysis were prioritized.

#### 2.1.3 Patient perspective

We conducted two focus group discussions and eight semi-structured interviews with individual patients undergoing HD or HDF and health care professionals in Germany, using a convenience sampling strategy. Inclusion criteria for patients were over 18 years, receiving maintenance dialysis therapy and able to speak and understand German. Inclusion criteria for health care professionals were having experience in treating patients on maintenance dialysis therapy (HD and HDF), and possessing the ability to communicate in German.

The focus of all interviews was on identifying health domains and symptoms relevant for patients undergoing maintenance dialysis therapy (i.e., HD or HDF). According to the aim of the CONVINCE trial, we were especially interested in those health domains and symptoms that could vary due to treatment modality.

We first conducted a focus group with five dialysis patients to gain better understanding of symptoms and problems relevant to patients. Subsequently, eight semi-structured interviews with individual patients were conducted to be able to address personal and sensitive issues, which might not be discussed in a focus group and to concentrate on the most relevant issues raised by individuals. In addition, we conducted one focus group interview with experienced health care professionals specialised in treating dialysis patients. This approach complemented the results of patient interviews and aimed to capture any potential additional issues not mentioned during patient interviews.

Participants were recruited at a university medical centre and two dialysis centres (one independent dialysis centre and one dialysis centre operated by a private dialysis service provider) in Berlin, Germany, between December 2017 and February 2018. All focus groups and interviews were audio-recorded and transcribed verbatim. Coding followed a directed content analysis approach informed by concepts identified in the literature review and core outcome sets. During analysis, the coding framework was iteratively refined when participant statements were not adequately represented by predefined categories. New categories were created inductively and reviewed within the research team until agreement was reached on whether they reflected distinct constructs, domains, or symptoms relevant to dialysis patients. The qualitative component was designed to support the identification and prioritization of relevant health domains and symptoms for toolbox development rather than to generate a standalone qualitative theory.

### 2.2 Triangulation and compilation of ESKD PRO toolbox

Concepts identified from the literature review, international outcome initiatives, and qualitative interviews were compiled into a master list and compared iteratively. Priority was given to concepts supported by multiple sources and considered highly relevant by patients. Concepts identified in only one source were retained only if they were judged clinically and conceptually important and feasible to assess in the CONVINCE trial setting. Domains were prioritized based on (i) recurrence across evidence sources, (ii) direct relevance to patients undergoing maintenance dialysis, (iii) anticipated sensitivity to treatment modality, and (iv) feasibility of repeated assessment within the CONVINCE trial.

### 2.3 Selection of PROMs for each health outcome included in the ESKD PRO toolbox

We investigated the coverage of the PROMIS^®^ health domain framework by matching them with concepts included in the ESKD PRO toolbox. Adequate coverage by PROMIS^®^ was defined as the availability of an established PROMIS^®^ domain whose conceptual content corresponded sufficiently to the prioritized health domain and could be implemented feasibly in the CONVINCE trial. PROMIS^®^ short forms were preferred over full item banks for routine administration because they reduced respondent burden, supported implementation across study sites and languages, and maintained scoring on a common metric. For prioritized concepts insufficiently covered by available PROMIS^®^ content—most notably dialysis-related fatigue and recovery—additional items were developed to capture treatment-related temporal patterns. Besides the reliability, validity and interpretability of the PROMs, practicable aspects related to the CONVINCE trial, such as language availability and respondent burden were taken into consideration (16). Newly developed items were intended for use in CONVINCE and will require further content and psychometric evaluation in subsequent work.

### 2.4 Ethical consideration

We performed all procedures of the study according to the Helsinki declaration and its later amendments. Ethical approval was obtained from the ethics committee at Charité Universitaetsmedizin Berlin, Germany. All participants consented to participate in the study.

## 3 Results

Results of the three consecutive steps developing the ESKD PRO toolbox are described hereinafter.

### 3.1 Conceptual considerations

The identification of health domains and symptoms were based on results of a literature review, core outcome sets and integration of the patient perspective.

#### 3.1.1 Targeted literature review

Based on the developed literature search strategy, we identified 295 reviews that considered PRO and PROMs in ESKD patients treated with maintenance dialysis therapy. After title abstract screening, 18 reviews were assessed in full text by the first author with 13 reviews included in the qualitative synthesis (overview of PRISMA Flow chart see **Figure 2**).

We identified 97 different PRO measures, which have been applied to ESKD patients in previous studies. We identified 26 measures assessing HRQL (generic, chronic generic and disease-specific). In addition, we identified measures to assess fatigue, sleep, pain, depression, anxiety, stress, social support, self-efficacy, self-worth, coping, emotional status, hope, illness severity, disease perception, health perception, functioning, sexual function, partnership, personality, treatment support, life satisfaction, stress, mood, religion, as well as measures to assess symptoms (**Supplementary Table 3**).

We extracted health domains and symptoms/problems covered by these measures. Symptoms were defined as patients’ experience of a deviation from what is perceived as normal, whereas health domains were defined as a set of related health functions or areas of life, which are subsumed to the multidimensional construct HRQL (25).

#### 3.1.2 Core outcome sets

Results of the COMET database revealed eight core outcome sets with two international initiatives were particularly relevant for the present work: the standard set for chronic kidney disease developed by the International Consortium for Health Outcomes Measurement (ICHOM), the Standardised Outcomes in Nephrology (SONG) initiative established a core outcome set for HD using internationally accepted and validated methods (26–28).

With regard to PROs, the ICHOM standard set included fatigue, pain, physical function and HRQL (27). The SONG-HD core outcomes set included 19 outcomes that we classified as potential PROs. These were fatigue, ability to travel, ability to work, depression, dialysis-free time, impact on family/friend, mobility, pain, washed out after dialysis, anxiety/stress, cognition, cramps, financial impact, food enjoyment, itching, nausea/vomiting, restless legs syndrome, sexual function, and sleep.

Concepts derived from these initiatives were compared with the literature-derived health domains and symptoms and subsequently reconciled with patient and healthcare professional interview findings. Where there was incomplete overlap, concepts supported across multiple sources and considered highly relevant to patients undergoing maintenance dialysis were prioritized.

#### 3.1.3 Patient perspectives

We included 13 patients (five men, eight women) undergoing HD or HDF in our study. All patients had been on dialysis between 3.5 months and 25 years. The focus group was conducted with five women aged between 25 and 86 years. The individual interviews with dialysis patients were conducted with eight patients (five men, three women) with a mean age of 54 years (ranging from 47–74 years). Further, we conducted a focus group with four health care professionals: two physicians, specialised in nephrology and two nurses (one with further education in dialysis care).

Fatigue emerged as one of the most salient health outcomes in patient narratives and was also recognized by healthcare professionals as highly relevant. Patients described fatigue as affecting physical function, cognitive function, and social participation, and often linked worsening fatigue to dialysis days and recovery afterwards. Sleep disturbance was also described as closely related to fatigue, including difficulties falling asleep, poor sleep quality, and repeated waking during the night. Patients emphasized variability in fatigue trajectories around dialysis sessions, including differences in onset, severity, and duration of recovery.

**Figure 3:**
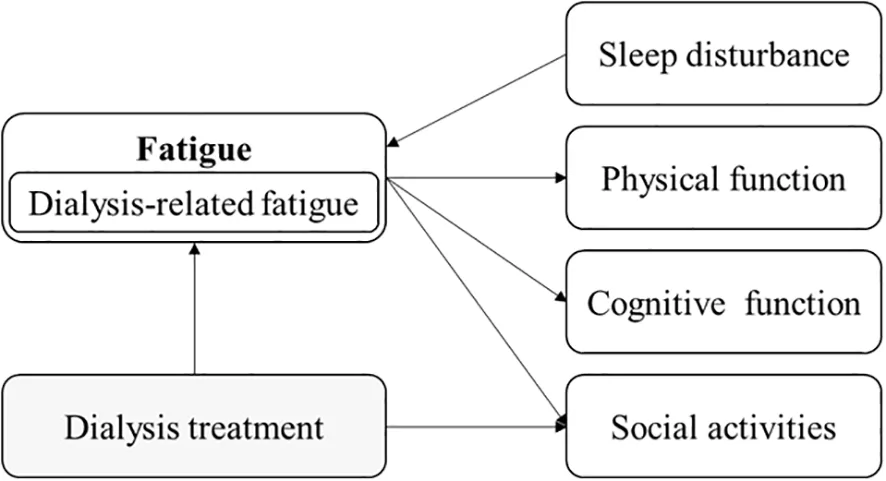
Relationships and dependencies of health domains reported by ESKD patients treated with maintenance dialysis. (n= 13).

Patients also reported a range of dialysis-related symptoms, including muscle cramps, dry and itchy skin, dry mouth and thirst, loss of appetite, nausea or upset stomach, blood pressure-related problems, and hot or cold spells. Anxiety and depression were discussed less frequently by patients but were retained as relevant domains because they were represented across evidence sources and judged important to patient wellbeing. Healthcare professional input was used to contextualize prevalence, perceived clinical relevance, and possible treatment sensitivity, but patient narratives remained the primary source for identifying salient domains and treatment-related experiences. **Figure 3** depicts the relationship and dependencies of selected health domains described by ESKD patients.

Among all patients, we were able to interview five patients currently treated with HDF, but had previous experience with HD. In this subgroup, we especially addressed if they perceived a difference in treatment in terms of frequency or intensity of symptoms or limitations in health domains. Three patients considered that the frequency and intensity of muscle cramps were less pronounced with HDF compared to HD. One patient stated that he felt more worn-out when he received HD and that his overall wellbeing had improved since starting HDF. These observations were treated as supportive contextual information rather than as formal comparative evidence.

### 3.2 Compilation of ESKD PRO toolbox

The list of health domains that emerged by reviewing the literature, and the list of concepts compiled, by reviewing core outcome sets were compared and reconciled with due consideration of the target population. This resulted in a draft list of eleven health domains and 41 disease-specific symptoms and problems considered relevant for patients with ESKD. An overview of the health domains and coverage by selected PROMs and international initiatives is provided in **Supplementary Table 3**.

Based on the results of the interviews with patients and healthcare professionals, we refined and reduced the number of health domains from eleven to nine. Patient satisfaction and encouragement of dialysis staff were excluded from our initial list, as those were considered to focus on patient-reported experience rather than patient-reported outcomes. For the domain fatigue, the interviews revealed different characteristics associated with fatigue: generic fatigue, dialysis-related fatigue and treatment-related time to recovery were described by patients.

For symptom assessment, we identified 44 different symptoms/problems related to ESKD in patients undergoing maintenance dialysis therapy. However, through the interviews with both patients and healthcare professionals we were able to reduce the number of symptoms/problems, to only 20 considered to be most relevant and to occur most frequently.

The final ESKD PRO toolbox included the following health domains and symptoms: general health/health perception, fatigue, physical function, social activity/social roles, depression, anxiety, pain, sleep disturbance, and cognitive functions. In addition, the following symptoms were included: soreness of muscles, chest pain, cramps, itchy skin, dry skin, shortness of breath, faintness or dizziness, lack of appetite, being washed out or drained, numbness in hands or feet, nausea or upset stomach, headaches, blurred vision, joint pain or stiffness, restless legs symptoms, hot or cold spells, problems with sex life, problems with vascular access site, feeling cold during dialysis, blood pressure problems during dialysis, blood pressure problems in days without dialysis.

### 3.3 Selection of PROMs to assess outcomes included in the ESKD PRO toolbox

In the final step, PROMs to assess the health domains and symptoms included in the ESKD PRO toolbox were selected. We compared the PROMIS^®^ health domain framework with the health domains included in the ESKD PRO toolbox and identified eight domains captured by the PROMIS^®^ health domain framework. For capturing the domain general health/health transition, items from the SF-36 were chosen. For symptoms relevant to patients with ESKD we compared the identified disease-specific measures that included symptom assessment with our list of most relevant symptoms. The included 20 different symptoms related to ESKD have been widely assessed in ESKD patients using the KDQOL-SF symptom list (9).

For the domain fatigue, the interviews with patients revealed that a generic fatigue measure might not be sufficient to assess fatigue levels in dialysis patients. as no information on dialysis-related trajectories of fatigue are assessed by generic measure. In particular, the time-related patterns when and for how long fatigue was experienced in connection with the dialysis treatment on a specific day varied to a large extent. To address this issue, we created additional items assessing peri/intra-dialytic fatigue (CONVINCE Dialysis Fatigue Scale (CDFS)), recovery time patterns CONVINCE Time to Recovery (CTTR-1) and CONVINCE Energy Peak (CEP-1) to assess treatment-related energy peaks (see **Supplementary Tables 4**, **5**). An overview of all health domains and the selected PROMs to assess these health domains is provided in **Table 1**.

**Table 1:** ESKD PRO toolbox, overview of PROMs by health domain.

Domain	Instrument	Number of items	Recall period	Rationale
Fatigue	PROMIS Fatigue customized short form + CDFS	16	7 days + dialysis-specific	Core patient priority and treatment-sensitive domain
Treatment-related recovery	CEP-1 + CTTR1	2	7 days	Treatment-sensitive domain
Physical function	PROMIS Physical Function – Short Form 4a	4	none	Captures impact on daily activities
Social Roles	PROMIS-Ability to participate in social Roles and Activities – Short Form 4a	4	7 days	Reflects social isolation risk and ability to maintain meaningful relationships
Depression	PROMIS Depression – Short Form 4a	4	7 days	Important for monitoring mental health burdens linked ESKD
Anxiety	PROMIS Anxiety – Short Form 4a	4	7 days	Important for monitoring mental health burdens linked ESKD
Pain	PROMIS Pain Interference – Short Form 4a + PROMIS Pain Intensity item	5	7 days	Evaluates multi-causal pain and its direct impact on daily functioning
Sleep	PROMIS Sleep Disturbance – Short Form 4a	4	7 days	Linked to fatigue and impacted by symptoms and treatment effects
Cognitive function	PROMIS Cognitive Function Abilities – customized 4 item short form	4	7 days	
General Health	SF-36 Global Health Items	2	current	Captures overall health perception
Symptoms	KDQOL-SF symptom list (modified and extended)	22	7 days	Captures the specific, multi-organ symptom burden and side effects directly caused by haemodialysis

SF-36, 36-Item Short Form Health Survey; KDQOL-SF, Kidney Disease Quality of Life- Short Form; PROMIS^®^, Patient-Reported Outcomes Measurement Information System^®^.

The final ESKD PRO toolbox was designed for repeated longitudinal assessment of patient-reported outcomes within the CONVINCE trial. It comprised PROMIS^®^-based assessment of eight prioritized domains (fatigue, physical function, ability to participate in social roles and activities, depression, anxiety, pain interference, sleep disturbance, and cognitive function), supplemented by general health items from the SF-36, a modified KDQOL-SF symptom list to capture dialysis-relevant symptoms, and additional items developed to assess dialysis-related fatigue, post-treatment recovery time, and treatment-related energy peaks. This modular composition allowed standardized domain assessment while preserving the ability to capture dialysis-specific experiences not adequately represented in generic PROM frameworks.

## 4 Discussion

In this paper, we described the development of the ESKD PRO toolbox administered in the CONVINCE trial using a domain-based approach. We identified health domains and symptoms that are pertinent to patients undergoing maintenance dialysis therapy and aligned them with the PROMIS^®^ health domains framework and other PROMs integrated into the ESKD PRO toolbox. In addition, PROMs assessing concepts that were assumed to be potential differentiators between the two treatments HD and HDF as assessed in the CONVINCE trial were included.

In clinical research, the availability or the recommendation of particular PROMs often defines the domains being measured, instead of the other way around, which is to first think of what to measure, followed by how to measure it (16, 29, 30). A conceptual framework that defines and determines aspects of interest given a certain objective in due consideration of a specific population is often neglected, despite the recommendations of the FDA and other guidelines based on interdisciplinary cooperation between medical, natural and social sciences (14, 31, 32).

Initiatives such as the OMERACT (33), ICHOM (27, 34) or SONG (28) strive to standardise health outcomes, including PROs, on an international level by consensus building. These initiatives focus on what to measure by identifying core outcome domains and propose how to measure these domains. This is a shift in perspective, moving away from an instrument-guided approach of measuring patient-relevant aspects to a domain-guided approach. The PROMIS^®^ health domain framework takes up this shift, providing 90 health domains, which facilitate interoperability and flexible compilation (20, 35). Although we were able to cover most health domains by use of the PROMIS^®^ framework, dialysis-related fatigue as well as dialysis-specific and disease-specific symptoms could not be adequately covered, and thus were complemented by use of other established disease-specific measures. In the case of dialysis-related fatigue, analyses of the CONVINCE data set will help to further understanding the additional insights generated by use of the CEP-1 CTTR-1, and the CDFS.

Further, the issue of comparability of PROs was considered within the ESKD PRO toolbox. Most PROMs do not allow for customization or flexible adaptation due to scoring or comparability issues (36). Hence, we decided to apply PROMIS^®^ measures for the domains included in the toolbox whenever possible (18). PROMIS^®^ item banks were developed using item response theory methods. Thus, items of an item bank can be exchanged, added or excluded without compromising comparability of scores of a domain (18). Further, it is possible to reduce respondent burden by using computer adaptive tests (21, 37). In doing so, patients answer an individual set of items including only items relevant to them. By using PROMIS^®^ measures this improves measurement precision, and reduces a potential floor and ceiling effect by measuring over a wider range (35).

Recent recommendations from the EuDial Working Group have highlighted both the growing importance of patient-reported outcomes in dialysis research and the substantial heterogeneity in instruments and methodologies currently used to assess health-related quality of life across studies (5). The consensus further notes that treatment-related differences may be observed in domains including physical functioning, cognitive function, pain interference, symptom burden, and social participation. These observations reinforce the need for a standardized, conceptually grounded approach to patient-reported outcome assessment and provide additional support for the PROMIS- health domain-based ESKD PRO toolbox proposed in the present work.

Another issue, which can be addressed by a domain-guided approach, is the multi-morbidity of patients. Most ESKD patients have additional co-morbidities such as diabetes mellitus and cardiovascular disease, which can also impact on physical functioning and fatigue (28, 38, 39). By applying disease-specific PROMs, e.g., a kidney disease-specific PROM and a heart disease-specific PROM, the same or similar health domains can be assessed, but adds an additional burden to the patient, while hardly obtaining any new information. Approaching patient-reported outcome assessments from a domain perspective, as we did with ESKD PRO toolbox, enables the extension by additional domains related to co-morbidities, but without burdening the patient.

The approach that we took to develop the ESKD PRO toolbox has some limitations. The literature review was restricted to systematic reviews published between 2000 and 2018 because toolbox development occurred during the planning phase of the CONVINCE trial. Consequently, more recent PROMs and implementation models developed after this review window may not be fully represented, which should be considered when interpreting the scope of the toolbox. Further, participants were recruited through convenience sampling in Germany and interviews were conducted only with German-speaking individuals. This might have introduced selection bias and may limit transferability of findings to other linguistic, cultural, and healthcare contexts. Accordingly, the toolbox should be viewed as patient-informed and internationally triangulated, but initially developed within a specific clinical and geographic context of the CONVINCE trial. The qualitative work was designed to support concept elicitation and prioritization for instrument selection as part of our triangulation approach rather than to generate a standalone explanatory theory. Patient narratives were the primary source for identifying salient health domains and treatment-related experiences. Healthcare professionals contributed complementary contextual information regarding the frequency of symptoms in routine care, their perceived clinical relevance, and the likelihood that some domains might vary by dialysis modality. We gave careful attention to international initiatives such as SONG and ICHOM, and discussed results from the literature review and interviews in our international study group. Finally, the choice of PROMs to assess the patient-centred outcomes might seem arbitrary and other researchers might have chosen other instruments. Yet, this is similar and comparable to previous approaches that have been developed over the years but proven useful. Our choice was based on measurement properties and consistency, alongside with the aim to apply the next generation of PROMs, i.e., the PROMIS^®^ health domain framework allows to use various instruments for a domain assessment, enabling an ongoing improvement of tools, without changing the metric to maintain comparability.

We argue that a more flexible approach is required to measure what matters to patients using PROMs and to meet various requirements according to the area of application. While in clinical research typically additional resources are available to capture the patient’s perspective, in clinical practice PROM assessments need to fit smoothly into routines considering aspects of patient burden and stuff burden. A modular approach may help to tailor PROM assessments to the needs of the patients, and to preferences of the health care provider to facilitate their usage in daily practice settings, such as indicating interventions to improve the well-being of the individual patient. One prerequisite to utilize real-world data evidence for conducting future large-scale studies.

The domain-guided approach to assess relevant health aspects of patients undergoing maintenance dialysis therapy offers a flexible composition of domains according to the field of application and purpose. The compilation of PROMIS^®^ health domains (translated and culturally validated for various countries) to the ESKD PRO toolbox facilitates measuring relevant health domains more precisely and across a wider range, while reducing respondent burden, thus providing an easier and better suited tool for long term monitoring of dialysis patients.

Within CONVINCE, the ESKD PRO toolbox was intended to support repeated assessment of treatment-related changes in patient-reported symptoms, functioning, and other aspects of health-related quality of life over time. Its purpose was not diagnostic screening or direct bedside decision support, but standardized longitudinal outcome assessment in a large multicentre randomized trial. Subsequent analyses of the CONVINCE dataset have demonstrated the utility of this approach for detecting differences in trajectories of patient-reported health status between treatment groups (40). Although, the ESKD PRO toolbox was developed in the context of the CONVINCE trial, its domain-based structure is not trial-specific and may be adapted for other dialysis studies or clinical care settings, provided that implementation requirements, language availability, and context-specific validation needs are considered.

## Data Availability

The datasets presented in this article are not readily available because the qualitative data (interview transcripts) generated during and analysed during the current study are not publicly available due to data privacy reasons but are available from the corresponding author on reasonable request. Requests to access the datasets should be directed to the corresponding author.
