# Supplementary material for: Assessment of patient perspectives in the CONVINCE trial in end-stage kidney disease - a domain-guided approach
Source: Front Nephrol. 2026 Aug 26;6:1767603. doi: 10.3389/fneph.2026.1767603 (PMC13553382; doi:10.3389/fneph.2026.1767603)
Supplement: Supplementary file 1 [file DataSheet1.docx]

Assessment of Patient Perspectives in the CONVINCE Trial in End-Stage Kidney Disease - a Domain-Guided Approach

Supplementary Material

# 1 Supplementary Tables

| **Table S1:** Literature search strategy | | |
| --- | --- | --- |
| **No** | **Concept** | **PubMed entry** |
| #1 | *Construct:*  QoL and PRO^1^ | (HR-PRO[tiab] OR HRPRO[tiab] OR HRQL[tiab] OR HRQoL[tiab] OR QL[tiab] OR QoL[tiab] OR quality of life[tiab] OR life quality[tiab] OR health index*[tiab] OR health indices[tiab] OR health profile*[tiab] OR health status[tiab] OR ((patient[tiab] OR self[tiab] OR child[tiab] OR parent[tiab] OR carer[tiab] OR proxy[tiab]) AND ((report[tiab] OR reported[tiab] OR reporting[tiab]) OR (rated[tiab] OR rating[tiab] OR ratings[tiab]) OR based[tiab] OR (assessed[tiab] OR assessment[tiab] OR assessments[tiab]))) OR ((disability[tiab] OR function[tiab] OR functional[tiab] OR functions[tiab] OR subjective[tiab] OR utility[tiab] OR utilities[tiab] OR wellbeing[tiab] OR well being[tiab]) AND (index[tiab] OR indices[tiab] OR instrument[tiab] OR instruments[tiab] OR measure[tiab] OR measures[tiab] OR questionnaire[tiab] OR questionnaires[tiab] OR profile[tiab] OR profiles[tiab] OR scale[tiab] OR scales[tiab] OR score[tiab] OR scores[tiab] OR status[tiab] OR survey[tiab] OR surveys[tiab]))) |
| #2 | *Population:*  Patients with ACKD | chronic kidney[tiab] OR chronic renal[tiab] OR kidney failure[tiab] OR renal failure[tiab] OR esrd[ti] OR “Renal Insufficiency”[mh:noexp] OR “Renal Insufficiency, Chronic”[mh] OR “Kidney Diseases”[majr:noexp] OR “Renal Insufficiency”[mh] OR ckd[tiab] OR esrd[tiab] OR end-stage kidney[tiab] OR endstage renal[tiab] OR end-stage renal[tiab] |
| #3 | *Treatment:*  Dialysis | “Renal Replacement Therapy”[majr:noexp] OR “Hemofiltration”[majr] OR dialysis therapy[tiab] OR haemofilt*[tiab] OR hemofilt*[tiab] OR renal replacement[tiab] OR haemodi*[tiab] OR dialy*[tiab] OR hemodi*[tiab] OR “Renal Dialysis”[mh] |
| #4 | *Type of study:*  Systematic reviews^2^ | (((systematic review[ti] OR systematic literature review[ti] OR systematic scoping review[ti] OR systematic narrative review[ti] OR systematic qualitative review[ti] OR systematic evidence review[ti] OR systematic quantitative review[ti] OR systematic meta-review[ti] OR systematic critical review[ti] OR systematic mixed studies review[ti] OR systematic mapping review[ti] OR systematic cochrane review[ti] OR systematic search and review[ti] OR systematic integrative review[ti]) NOT comment[pt] NOT (protocol[ti] OR protocols[ti])) NOT MEDLINE [subset]) OR (Cochrane Database Syst Rev[ta] AND review[pt]) OR systematic review[pt] |
| #5 | #1 AND #2 AND #3 AND filters | Filters Publication date from 2000/01/01 to 2018/12/31; Humans; English |
| QoL: Quality of Life; PRO: Patient-Reported Outcomes; ESKD: End-stage kidney disease  ^1^Modified search filter by Gibbons E et al. 2010, <https://www.cosmin.nl/wp-content/uploads/prom-search-filter-oxford-2010.pdf>  ^2^ PubMed Systematic Reviews Filter 2018 <https://www.nlm.nih.gov/bsd/pubmed_subsets/sysreviews_strategy.html> | | |

| **Table S2:** PICOS tool, used for study selection of literature review | |
| --- | --- |
| **Criteria** | **Inclusion criteria** |
| Population | Person ≥ 18 with end-stage kidney disease undergoing maintenance dialysis therapy |
| Intervention | No restrictions |
| Comparison | No restrictions |
| Outcome | Quality of life, patient reported outcomes |
| Study type | Systematic literature review |
| Other | English language |

| **Table S3: Overview of PROMs applied in ESKD patients** | | | |
| --- | --- | --- | --- |
| **Domain** | **PROM - abbreviation** | **PROM -** | **Descriptions** |
| Anxiety | STAI | State-Trait Anxiety Inventory | A 40-item measure including 20 items assessing trait anxiety and 20 for state anxiety. |
| Anxiety | BAI | Beck Anxiety Inventory | A 21-item measures assessing common symptoms of anxiety. |
| Coping | PAIS | Psychological Adjustment to Illness Scale | A-46-item to assess the quality of a patient’s psychosocial adjustment to a medical condition covering aspects of health care orientation, vocational environment, domestic environment, sexual relationships, extended family relationships, social environment, and psychological distress. |
| Coping | WCCL | Ways of coping checklist | A 66-item measures to assess thoughts and acts used to deal with the internal and/or external demands of specific stressful encounters. |
| Coping | Brief COPE | Brief COPE | A 28-item measures to assess effective and ineffective ways to cope with a stressful life event covering aspects of self-distraction, active coping, denial, substance use, use of emotional support, use of instrumental support, behavioural disengagement, venting, positive reframing, planning, humour, acceptance, religion, and self-blame. |
| Coping | JCS | Jalowiec Coping Scale | A-60-item measure to assess situation-specific coping behaviour by measuring the degree of use and the perceived effectiveness of cognitive and behavioural coping strategies in a stressful situation designated by the researcher or clinician. |
| Depression | CES-D | Center for Epidemiologic Studies Depression Scale | A 20-item measure to assess various symptoms of depression focusing on affective component of depression. |
| Depression | PHQ-9 | Patient Health Questionnaire - 9 | A 9-item measure to assess presence and severity of depression considering DSM-IV criteria for the diagnosis of major depression. |
| Depression | HDRS | Hamilton Depression Rating Scale | A 17-item measure to assess severity of, and change in depressive symptoms covering suicide, depressed mood, feelings of guilt, insomnia work and activities, retardation, agitation, anxiety psychic/somatic, insight, loss of weight, hypochondriasis, genital symptoms and somatic symptoms. |
| Depression | BDI | Beck Depression Inventory | A 21-item measure to assess the severity of depressive symptoms in the clinical setting. |
| Depression | BDI-II | Beck Depression Inventory-II | A 21-item measure to assess psychological and somatic manifestations of major depressive episodes as operationalized in the DSM-IV. |
| Depression | CDI | Cognitive Depression Index | A 15-item measure that is a subscale of the 21-question Beck Depression Inventory (BDI) excluding items related to somatic symptoms. |
| Depression | SDS | Zung Self-rating Depression Scale | A 20-item measure to assess depression covering four common characteristics of depression: the pervasive effect, the physiological equivalents, other disturbances, and psychomotor activities. |
| Depression | GDS | Geriatric Depression Scale | A 30-item measure to assess depression in the elderly. |
| Depression | HADS | Hospital Anxiety and Depression Scale | A 14-item measure to assess anxiety and depression. Seven of the items relate to anxiety and seven relate to depression. |
| Depression | DASS-21 | Depression, Anxiety and Stress Scale | A 21-item measure to assess the emotional states of depression, anxiety and stress. The depression scale assesses dysphoria, hopelessness, devaluation of life, self-deprecation, lack of interest / involvement, anhedonia and inertia. The anxiety scale assesses autonomic arousal, skeletal muscle effects, situational anxiety, and subjective experience of anxious affect. The stress scale assesses difficulty relaxing, nervous arousal, and being easily upset / agitated, irritable / over-reactive and impatient. |
| Disease perception | IEQ | Illness Effects Questionnaire | A 20-item scale to assess the patient’s perception about the global effect of the illness and covers biological psychological, and social aspects associated with becoming ill. |
| Disease perception | IPQ | Illness Perception Questionnaire | A 38-item measure to assess cognitive representations of illness covering the 5 domains identity scale, time-line, consequences, cure control, and cause. |
| Disease severity | ESRD-SI | End Stage Renal Disease Severity Index | A 1-item scale to evaluate the severity of illness in a patient with end-stage kidney disease using 6-Point Likert scale. |
| Disease-related loss | KDLS | Kidney Disease Loss Scale | A 6-item measure to assess the level of cognitive, affective responses and dialysis-related losses of patients with ACKD. |
| Distress | IES-R | Impact of Events scale - revised | A 22- item measure to assess subjective distress caused by traumatic events |
| Fatigue | BFI | Brief Fatigue Inventory | A 9-item measure to assess the severity and impact of fatigue including impact on general activity, mood, walking ability, normal work, relations with other people and enjoyment of life. |
| Fatigue | CFQ | Chalder Fatigue Scale | A 11-item measure assessing the severity of tiredness in fatiguing illness related to physical and mental fatigue. |
| Fatigue | FACIT-Fatigue | Functional Assessment of Chronic Illness Therapy - Fatigue, Fatigue Impact Scale | A 13-item measure to assess level of fatigue during usual daily activities. |
| Fatigue | VAS-Fatigue Severity | Visual Analogue Scale to Evaluate fatigue Severity | An 18-item measure to assess subjective experience of severity of fatigue and energy. |
| Fatigue | RFS | Rhoten Fatigue Scale | A 1-item scale to rate fatigue level on an 11-point numerical rating scale. |
| Fatigue | PFS | Piper Fatigue Scale | A 22-item measure to assess fatigue and its impact on behaviour, affective, sensory, and cognitive/mood. |
| Fatigue | MAF | Multidimensional Assessment of Fatigue | A 16-item measure to assess fatigue according to four dimensions: degree and severity, distress, timing of fatigue (over the past week, when it occurred and any changes), and its impact on various activities of daily living (household chores, cooking, bathing, dressing, working, socializing, sexual activity, leisure and recreation, shopping, walking, and exercising). |
| Fatigue | MFI | Multidimensional Fatigue Inventory | A 20-item measure to assess fatigue covering the following dimensions: General Fatigue, Physical Fatigue, Mental Fatigue, Reduced Motivation and Reduced Activity. |
| Fatigue | FSS | Fatigue Severity Scale | A 9-item measure to assess fatigue and its effect on a person's activities and lifestyle in patients with a variety of disorders. |
| Fatigue | FSI | Fatigue Symptom Inventory | A 14-item measure to assess the severity, frequency, and daily pattern of fatigue as well as its perceived interference with quality of life (general level of activity, ability to bath and dress, normal work activity, ability to concentrate, relations with others, enjoyment of life, and mood). |
| Functioning | COOP/WONCA-charts | COOP Functional Health Assessment Charts/WONCA | A 6-item measure to assess core aspects of functional status including physical fitness, feelings, daily activities, social activities, change in health and overall health. In addition, pain can be included as an optional aspect. |
| Health perception | SIP | Sickness-Impact Profile | A 136-item measure to assess a person's perception of their health status with respect to their disease impact. It covers 12 dimensions: sleep and rest, eating, work, home management, recreation and pastimes, ambulation, mobility, body care and movement, social interaction, alertness behaviour, emotional behaviour and communication |
| Hope | THS | Trait Hope Scale | A 12-item measure to assess the extend of hope covering aspects of pathways thinking and agency thinking. |
| HRQL | Agarwal | Agarwal | A 37-item HRQL measure for use in non-dialysis patients with mental and physical dimensions. |
| HRQL | KDQOL | Kidney Disease Quality of Life | A 134-item measure to assess health-related quality of life designed for use in kidney disease patients undergoing dialysis. Besides the 8 dimension of the SF-36, 8 disease-specific dimensions are covered including 'symptoms/problems', 'effects of kidney disease on daily life', 'burden of kidney disease', 'work status', 'cognitive function', 'quality of social interaction', 'sexual function', 'sleep'. Three additional dimensions are included in the measure, 'social support', 'dialysis staff encouragement', 'patient satisfaction'. |
| HRQL | KDQOL-SF | Kidney Disease Quality of Life - short form | An 80-item HRQL measure designed for use in kidney disease patients undergoing dialysis. Besides the SF-36, 8 disease-specific dimensions are covered including 'symptoms/problems', 'effects of kidney disease on daily life', 'burden of kidney disease', 'work status', 'cognitive function', 'quality of social interaction', 'sexual function', 'sleep'. Three additional dimensions are included in the measure, 'social support', 'dialysis staff encouragement', 'patient satisfaction'. |
| HRQL | KDQOL-36 | Kidney Disease Quality of Life - 36 | A 36-item HRQL measure designed for use in kidney disease patients undergoing dialysis. It is derived from the KDQOL-SF including the 2 disease specific dimensions 'symptoms and problems', 'burden of kidney disease' and 'effects of kidney disease'. Further the SF 12 is included to assess generic HRQL. |
| HRQL | CDQOL | Chinese Dialysis Quality of Life Scale | A 29-item measure to assess QoL of Chinese dialysis patients |
| HRQL | CHEQ | CHOICE Health Experience Questionnaire | An 83-item measure to assess HRQL, and the effectiveness of dialysis modalities. Besides the 8 dimensions of the SF-36, it includes 13 dialysis-specific dimensions: freedom, travel restrictions, cognitive functioning, financial, restrictions on diet and fluids, recreation, work, body image, symptoms, sleep, sexual functioning, access-related problems, quality of life. |
| HRQL | KDQ | Kidney Disease Questionnaire | A 26-item measure to assess effects of intervention on the QOL of patients undergoing haemodialysis including the 5 dimensions: physical symptoms, fatigue, depression, relationship with others, frustration. |
| HRQL | WHOQOL-BREF (Dialysis) | World Health Organisation Quality of Life - Brief | A 26-item measure covering the domains: physical health, psychological health, social relationships and environment. The measure also includes two global items (general QOL and general health). |
| HRQL | QLI | Quality of Life Index 3.0 | A 68-item QOL measure including additional dialysis-related items covering the domains health and functioning, social economic, psychological/spiritual, family. Satisfaction with various domains of life as well as the importance of the domain to the individual are assessed. |
| HRQL | KTQ | Kidney Transplant Questionnaire | A 25-item measure for use in kidney transplant patients covering the 5 dimensions, physical symptoms, fatigue, uncertainty/fear, appearance, emotional. |
| HRQL | RTQ V1 | ReTransQOL version 1 | A 45-item measure designed to assess QoL in renal transplant patients covering the 5 dimensions physical health, mental health, medical care, fear of losing graft, treatment. |
| HRQL | RTQ V2 | ReTransQoL version 2 | A 32-items measuring QoL in renal transplant patients including the 5 domains physical health, social functioning, medical care, treatment, fear of losing graft. |
| HRQL | GIQLI | Gastrointestinal Quality of Life Index | A 36-item measure focussing on the impact GI complaints on HRQL of renal transplant patients covering the 5 dimensions GI symptoms, emotional status, physical function, social function, strain of medical treatment. |
| HRQL | NHP | Nottingham Health Profile | A 38-items measure to assess generic HRQL grouped into the 6 dimensions pain, energy, physical mobility, sleep, emotional reactions, social isolation. |
| HRQL | SF-36 | Health Survey Short Form 36 Items | A 36-items to assess generic HRQL covering the 8 domains physical functioning, physical role, bodily pain, general health, vitality, social functioning, emotional role, mental health and one additional item to measure self-evaluated change in health status. |
| HRQL | SF-12 | Health Survey Short Form 12 Items | A 12-item measures derived from the SF-36, covering 8 dimensions that are computed into 2 distinct clusters (Physical Component Score and Mental Component Score). |
| HRQL | EQ-5D | EuroQol - 5 Dimension | A utility measure with 5 dimensions mobility, self-care, usual activities, pain/discomfort, anxiety/depression, plus VAS assessing overall health status. |
| HRQL | SQLI | Spitzer Quality of Life-Index | A 5-items measure to assess QOL covering the 5 dimensions activity, daily living, health, support, and future perspective. |
| HRQL | EORTC- QLQ-C30 | European Organisation for Research and Treatment in Cancer Quality of Life questionnaire Core 30 | A 30-item measures to assess HRQL in oncological patients covering 5 functional scales (physical functioning, role functioning, emotional functioning, cognitive functioning, social functioning) and 9 symptoms scales (fatigue, nausea and vomiting, pain, dyspnoea, insomnia, appetite loss, constipation, diarrhoea, financial difficulties) and a global health status |
| HRQL | SWED-QUAL | Swedish Health-Related Quality of Life Survey | A 61-item measure (adapted from the Medical Outcomes Study) including 11 multi-item scales covering aspects of physical, mental, social, and general health. |
| HRQL | QWB / QWB-SA | Quality of well-being / Quality of Well Being Self-administered Scale | A 71-item measure to assess overall health status and well-being in the four area physical activities, social activities, mobility and symptom/problem complexes. |
| HRQL | MQOL | McGill QoL questionnaire | A 17-item measure to assess physical well-being, physical symptoms, psychological symptoms, existential well-being and support as well as overall quality of life. |
| HRQL | GHQ-28 | General Health Questionnaire (28 Items Version) | A 28-item measures to assess inability to carry out normal functions and the appearance of new and distressing phenomena, screening for non-psychotic psychiatric disorders. |
| HRQL | QUAL-E | Quality of Life at the End of Life | A 25-item measure that cover the 4 domains: symptom impact, relationship with healthcare provider, preparation for end of life, life completion. |
| HRQL | Ferrans and Powers QLI | Ferrans and Powers QOL Index-dialysis version | A 68-item measure to assess satisfaction and importance of certain aspects of life covering the dimensions: health and functioning, psychological /spiritual, social and economic, family and overall quality of life. |
| HRQL | IBDQ | Inflammatory Bowel Disease Questionnaire | A 32-item measure to assess quality of life of patients with inflammatory bowel diseases covering bowl symptoms, emotional health, systemic symptoms and social functioning. |
| Insomnia | DISS | Daytime Insomnia Symptom Scale | A 20-item measure to assess daytime functioning including subjective vitality, mood, and alertness. |
| Life satisfaction | SWLS | Satisfaction with life scale | A 5-item measure to assess global cognitive judgement of one's life satisfaction of the respondent' life as a whole. |
| Life satisfaction | LSI | Life Satisfaction Index | A 20-item measure to assess general feelings of well-being among older people to identify “successful” aging. Subdomains: zest (as opposed to apathy), resolution and fortitude, congruence between desired and achieved goals, positive self-concept and mood tone. Positive well-being is indicated by the individual taking pleasure in his daily activities, finding life meaningful, reporting a feeling of success in achieving major goals, a positive self-image and optimism. |
| Mood | POMS | Profile of Mood States | A 65-item measure covering tension-anxiety, depression, anger-hostility, fatigue, confusion-bewilderment, and vigour-activity |
| Pain | MPQ | McGill Pain Questionnaire | A 20-item measure to assess the quality and intensity of pain covering the domains: sensory, affective, evaluative, and miscellaneous. |
| Partnership | DAS | Dyadic Adjustment Scale | A 32-item measure to assess couple satisfaction and to evaluate how each partner within the couple perceives his or her relationship including aspects related to sex life, fights, commitment, household, Agreement in different topics, general happiness. |
| Religiosity | Hoge IR Scale | Hoge Intrinsic Religiosity Scale | A 10-item measure to assess religiosity covering aspects of religious beliefs or experiences. |
| Restless leg syndrome | RLS-RS | Restless Leg Syndrome Questionnaire (Rating Scale) | A 6-item measures to assess severity of restless leg syndrome. |
| Self-efficacy | SUPPH | Strategies Used by People to Promote Health | A 29-item measure to assess self-care self-efficacy covering aspects of coping, stress reduction, making decisions, and enjoying life. |
| Self-efficacy | GSE | General Self-Efficacy Scale | A 10-item measure to assess a general sense of perceived self-efficacy to predict coping with daily hassles as well as adaptation after experiencing all kinds of stressful life events. |
| Self-efficacy / locus of control | MHLC | Multidimensional Health Locus of Control Scale | An 18-item measure to assess health locus of control covering aspects of internal, external chance, and external powerful others. |
| Self-esteem | RSES | Rosenberg Self-esteem Scale | A 10-item measures to assess self-esteem covering aspects of both positive and negative feelings about the self. |
| Sexual function | IIEF | International Index of Erectile Function | A 15-item measure to assess the 4 main domains of male sexual function: erectile function, orgasmic function, sexual desire and intercourse satisfaction. |
| Sleep | PSQI | Pittsburgh Sleep Quality Index | A 19-item measure to assess sleep quality and disturbance covering aspects of subjective sleep quality, sleep latency, sleep duration, habitual sleep efficiency, sleep disturbances, use of sleep medication, and daytime dysfunction. |
| Sociability | EPQ-R | Eysenck Personality Questionnaire-revised | A 100- item measure to assess three major dimensions of personality, i.e. extraversion/introversion, neuroticism, and psychoticism or tough-mindedness. |
| Social engagement | LSNS | Luben Social Network Scale | A 12-item measure to assess social engagement, focusing on social networks of family and friendship. |
| Social support | SSA | Social Support Apgar | The Social Support Apgar (SSA), developed as a user-friendly tool for screening for perceptions of adequacy of social support among perinatal woman, covering perceived satisfaction with different types of support. |
| Social support | ISEL | Interpersonal Support Evaluation List | A 40-item scale to assess perceived availability of four types of social support covering aspects of appraisal, belonging, self-esteem, and tangible support. |
| Stress | HSS | Haemodialysis stress scale | A 29-items to assess physiological and psychological stressors in haemodialysis patients. |
| Stress | PSS-10 | Perceived Stress Scale -10 | A 10-item measure to assess the perception of stress, including current level of experienced stress and degree of situations in one’s life appraised as stressful |
| Stress | PSS-4 | Perceived Stress Scale - 4 | A 4-item measure to assess the perception of stress, including current level of experienced stress and degree of situations in one’s life appraised as stressful |
| Symptom | modified ESAS | Modified Edmonton Symptom Assessment System | A 9-item measures to assess common symptoms among patients with advanced cancer, covering pain, tiredness, nausea, depression, anxiety, drowsiness, appetite, feeling of wellbeing and shortness of breath including space to add an additional optional symptom. |
| Symptom and treatment-related impact | ESRD-SCL-TM | End-Stage Renal Disease Symptom Checklist - Transplantation Module | A 43-item measure to assess limited physical capacity, limited cognitive capacity, cardiac and renal dysfunction, side effects of corticosteroids, increased growth of gum and hair, transplantation-related psychological distress in renal transplant patients on immunosuppression. |
| Symptom burden | CKD-SBI | CKD-Symptom Burden Index | A 32-item measure to assess symptom burden covering the dimensions: prevalence, distress, severity, frequency used in patients with CKD stages IV and V. |
| Symptoms | PSA | Parfrey Symptom Assessment | The measure assesses the symptom tiredness, cramps, pruritus, dyspnoea, headaches and joint pain in terms of different symptom attributes, i.e., severity, frequency, duration, effect on sleep, daily living, activity, subjective quality of life and necessity for drug therapy. |
| Symptoms | GSRS | Gastrointestinal Symptom Rating Scale | A 15-item measure to assess important gastrointestinal side effects of immunosuppressive therapy in renal transplant patients covering the 5 dimensions: reflux, diarrhoea, constipation, abdominal pain, indigestion. |
| Symptoms | DSI | Dialysis Symptom Index | A 30-item measures to assess physical and emotional symptoms burden in patients in dialysis patients and their severity. |
| Symptoms | SSI | Somatic Symptoms Index | The Somatic Symptom Index has been derived from the Diagnostic Interview Schedule's somatization disorders items. It is a screening instrument for somatoform disorders. |
| Symptoms | RSCL | Rotterdam Symptom Checklist | A 30-item measures to assess symptoms of cancer patients, covering the four domains, physical symptom distress, psychological distress, activity level and overall global life quality. |
| Symptoms | SDS | Symptom Distress Scale | A 13-item measure to assess the degree of discomfort from the specific symptoms being experienced, as perceived by the patient. |
| Symptoms | ESRD-SCL | ESRD Symptom Check List | A 43-item measure to assess symptoms covering domains impaired physical performance, impaired cognitive performance, cardiac and renal dysfunction, cortisone side effects, increased hair and gum growth, and transplant-related mental stress |
| Symptoms | MSAS-SF | Memorial Symptom Assessment Scale Short Form | A 32-item measure to assess symptoms with respect to distress or frequency. A physical symptom subscale, psychologic symptom subscale, and global distress index can be derived from the Short Form. |
| Symptoms | PSDS | Physical Symptom Distress Scale | A 16-item measures to assess physical symptoms of ACKD patients undergoing haemodialysis. |
| Symptoms & palliative care | POS | Palliative Care Outcome Scale Symptoms (renal) | A 11-item measure assessing the most common symptoms renal patients experience plus additional items on concerns beyond symptoms, such as information needs, practical issues, family anxiety. |
| Symptoms + heath concerns | POS | Palliative Care Outcome Symptom Scale | A 11-item measure to assess physical and psychological symptoms, spiritual considerations, practical concerns, emotional concerns and support needs. The POS is completed by staff and by the patient. |
| Treatment satisfaction | RTSQ | Renal Treatment Satisfaction Questionnaire | A 11-item measure to assess satisfaction with treatment for patients with chronic kidney disease. It includes aspects such as side effects, treatment demands, convenience, flexibility, and understanding renal treatment. |
| Treatment-related symptoms | MTSOSDS | Modified Transplant Symptom Occurrence and Symptom Distress Scale | A 45-item measure to assess transplantation-related symptoms associated with the use of immunosuppression therapy. |

| Table S4: Items to assess treatment-related energy peak (CONVINCE Energy Peak (CEP-1) and time-to-recovery (CONVINCE Time to Recovery (CTTR-1)) | |
| --- | --- |
| *Item* | *Responses* |
| During the past 7 days, …  When did you experience the highest level of energy? | Dialysis day: before dialysis,  Dialysis day: during dialysis,  Dialysis day: evening,  Day after dialysis: morning,  Day after dialysis: afternoon |
| During the past 7 days, …  How much time did you need on average to recover from dialysis? | Less than 2 hours,  2-4 hours,  5-7 hours,  8-12 hours,  More than 12 hours |

| **Table S5:** Items to assess peri-/intra-dialytic fatigue (CONVINCE Dialysis Fatigue Scale (CDFS)) | | |
| --- | --- | --- |
| Item | | Responses |
| Dialysis day | I feel worn out before dialysis. | Not at all, a little bit, somewhat, quite a bit, very much |
|  | I feel worn out during dialysis. | Not at all, a little bit, somewhat, quite a bit, very much |
|  | I feel worn out in the evening on dialysis days. | Not at all, a little bit, somewhat, quite a bit, very much |
| Day after dialysis | I feel worn out in the morning the next day. | Not at all, a little bit, somewhat, quite a bit, very much |
|  | I feel worn out in the afternoon the next day. | Not at all, a little bit, somewhat, quite a bit, very much |
| Dialysis day | I have energy before dialysis. | Not at all, a little bit, somewhat, quite a bit, very much |
|  | I have energy during dialysis. | Not at all, a little bit, somewhat, quite a bit, very much |
|  | I have energy in the evening on dialysis days. | Not at all, a little bit, somewhat, quite a bit, very much |
| Day after dialysis | I have energy in the morning the next day. | Not at all, a little bit, somewhat, quite a bit, very much |
|  | I have energy in the afternoon the next day. | Not at all, a little bit, somewhat, quite a bit, very much |
